# Patterned Immobilization of Antibodies within Roll-to-Roll Hot Embossed Polymeric Microfluidic Channels

**DOI:** 10.1371/journal.pone.0068918

**Published:** 2013-07-18

**Authors:** Belachew Feyssa, Christina Liedert, Liisa Kivimaki, Leena-Sisko Johansson, Heli Jantunen, Leena Hakalahti

**Affiliations:** 1 VTT Technical Research Centre of Finland, Oulu, Finland; 2 Department of Forest Products Technology, School of Chemical Technology, Aalto University, Espoo, Finland; 3 Microelectronics and Materials Physics Laboratories, Department of Electrical Engineering, University of Oulu, Oulu, Finland; University of Houston, United States of America

## Abstract

This paper describes a method for the patterned immobilization of capture antibodies into a microfluidic platform fabricated by roll-to-roll (R2R) hot embossing on poly (methyl methacrylate) (PMMA). Covalent attachment of antibodies was achieved by two sequential inkjet printing steps. First, a polyethyleneimine (PEI) layer was deposited onto oxygen plasma activated PMMA foil and further cross-linked with glutaraldehyde (GA) to provide an amine-reactive aldehyde surface (PEI-GA). This step was followed by a second deposition of antibody by overprinting on the PEI-GA patterned PMMA foil. The PEI polymer ink was first formulated to ensure stable drop formation in inkjet printing and the printed films were characterized using atomic force microscopy (AFM) and X-ray photoelectron spectroscopy (XPS). Anti-CRP antibody was patterned on PMMA foil by the developed method and bonded permanently with R2R hot embossed PMMA microchannels by solvent bonding lamination. The functionality of the immobilized antibody inside the microfluidic channel was evaluated by fluorescence-based sandwich immunoassay for detection of C-reactive protein (CRP). The antibody-antigen assay exhibited a good level of linearity over the range of 10 ng/ml to 500 ng/ml (R^2^ = 0.991) with a calculated detection limit of 5.2 ng/ml. The developed patterning method is straightforward, rapid and provides a versatile approach for creating multiple protein patterns in a single microfluidic channel for multiplexed immunoassays.

## Introduction

The analysis of biomarkers directly at the side of a patient, which is known as point-of-care testing (POCT), is a continuously expanding trend in the practice of medical diagnosis [1]–[3]. Many of the available POCT devices on the market are based on immunoassays. Among the various immunoassay formats, enzyme-linked immunosorbent assay (ELISA) is a long standing-standard for the quantitative analysis of several diseases biomarker because of its sensitivity and specificity [4]. However, the conventional ELISA technique is often subjected to long incubation times and multiple washing steps, which limit its throughput and applicability for rapid biomarker testing. Miniaturization of conventional assays into POCT devices provides several advantages, such as simplification of the assay procedures, portability, reduced assay time, and lower consumption of samples and reagents. Several types of immunoassay devices have been developed and commercialized for POCT applications [5], [6]. Among these devices, the lateral flow-based pregnancy test, in which an antigen is detected to be above a certain threshold, is the simplest and most commercialized POCT device. Although this type of test is simple to perform, the result is not reproducible, quantitative and sensitive [2], [7]. Microfluidic devices are better options for testing target analytes that require quantification with less sample volume and increased sensitivity and accuracy [2], [6]-[9].

In parallel with the analytical need of microfluidic immunoassay systems for POCT applications, there is a large demand for fabrication of future devices used for POCT as inexpensive and disposable platform [10]. Silicon and glass are the commonly used substrates for fabrication of microfluidic devices. However, the expensive and time-consuming fabrication process limits the practical applicability of those materials for commercial immunoassays. Recently, there has been an increasing interest in the use of polymer and plastic materials for fabrication of microfluidics because of their mechanical, optical and chemical stability, low production cost and excellent processing properties. Poly (dimethylsiloxane) (PDMS) is a widely used elastomeric polymer in academic research for rapid prototyping of microfluidics via soft lithographic techniques because of its desirable optical properties, flexibility, and cost-effectiveness. However, the inherent limitations of PDMS for POCT applications include its hydrophobicity, propensity for protein adsorption and difficulties in scaling up for mass production [11]. Currently, thermoplastic materials, such as polymethyl methacrylate (PMMA), cyclic olefin copolymer (COC), polycarbonate (PC), polypropylene (PP), and polystyrene (PS), are increasingly used for extremely high-volume production of microfluidics using R2R hot embossing and injection molding [12]. Compared to injection molding, R2R hot embossing is a fast production method for microfluidic devices in which a rotating embossing cylinder transfers its stamp features onto a heated polymer web [12], [13].

Several approaches have been implemented to immobilize and pattern biomolecules on the surface of microchannels, including microcontact printing [14], [15], microfluidic patterning [16], photolithography [17], physical entrapment [18], deposition through stencil [19] and inkjet printing [20]. Among these methods, inkjet printing has multiple advantages such as simplicity, flexibility, low-cost of process, minimum consumption of materials and the capability to pattern multiple analytes simultaneously using independent cartridges. Piezoelectric inkjet printing has been applied to deposit antibodies on streptavidin-coated glass slides [20], EDC/NHS activated nanofibrillar cellulose films [21], nylon membranes [22], aldehyde functionalized silicon substrates [23] and streptavidin-coated gold surfaces [24]. The major methods used for the immobilization of biomolecules onto solid surfaces are physical adsorption by electrostatic force on charged surfaces or by hydrophobic interactions, physical entrapment, receptor/ligand pairing and covalent immobilization. Among these techniques, covalent immobilization offers several advantages by providing the most stable bond between the biomolecule and functionalized surfaces [15], [17], [25]. Particularly, immobilization by covalent bonding is desirable for creating a wash-stable protein pattern in microfluidic systems operating under flow conditions, as fluid movement promotes desorption of molecules from surfaces [17].

Poly (methyl methacrylate) (PMMA) is a thermoplastic polymer well-suited for roll-to-roll hot embossing of microfluidic devices for bioanalytical application because of its high transparency, low background fluorescence, low glass transition temperature (Tg = 105°C) and relatively low cost (less than 6 $US per square meter). However, PMMA has no specific functional group that binds biomolecules covalently [26]. Therefore, this polymer’s surface must be modified using chemical linkers to allow binding between the substrate and the biological interface. PEI is a polymer containing a high density of amine groups that can readily form covalent bonds with bifunctional chemical linkers such as glutaraldehyde to create amine reactive aldehyde surface on various solid surfaces. This polymer has been used to immobilize cells, DNA and proteins on various solid surfaces [27]–[29]. In addition, PEI is known to be immobilized covalently on PMMA surfaces through spontaneous adsorption from basic aqueous solutions [29], [30]. Bai et al. [29] coated PMMA microchannel surfaces with branched PEI by a dip-coating technique to immobilize antibodies covalently on the channel surface through glutaraldehyde crosslinking.

**Figure 1:**
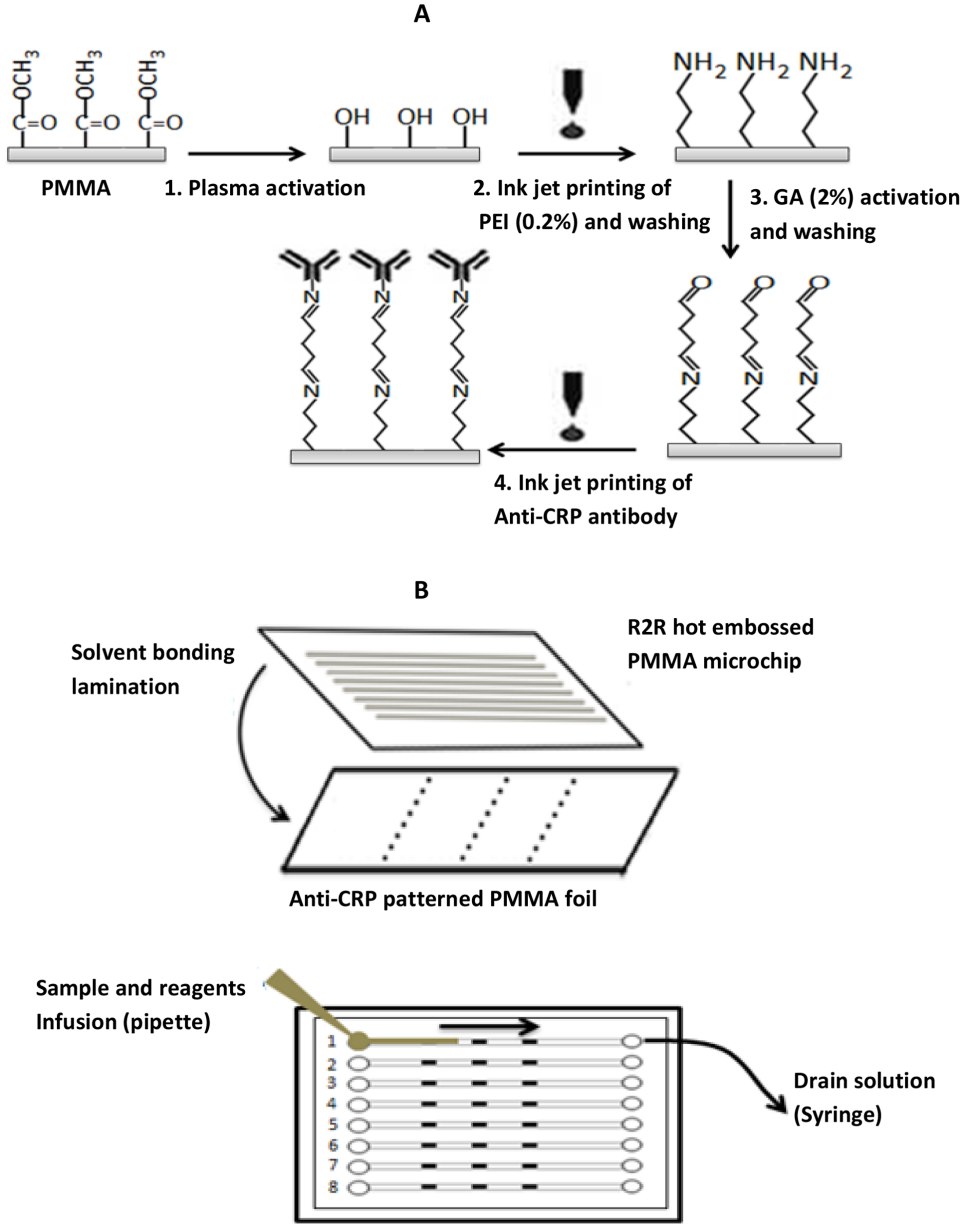
Inkjet printing of antibody array and assay format. (A) Schematic of the two-stage antibody patterning using the PEI and GA cross linker system. PEI ink was first deposited by piezoelectric inkjet printing in discrete regions of a PMMA foil. After activating the PEI patterned surface with glutaraldehyde, anti-CRP antibody was deposited locally in a second inkjet printing step. (B) A multichannel R2R hot embossed PMMA microfluidic chip is aligned and bonded permanently with the antibody patterned PMMA foil by solvent bonding lamination. Blocking reagent, mixture of sample and detection antibody, and wash buffer are sequentially introduced at one end of the microchip by pipette and drawn using syringe.

In this study, the above-mentioned covalent linkage chemistry was used and the immobilization process was optimized for the large-scale fabrication of antibody patterned foil-based immunoassay chips with printing method. The printing process includes sequential inkjet printing of both the PEI and antibody layers to achieve high-quality, wash-stable and customizable antibody patterns in a PMMA microfluidic platform. Our methodology is outlined in Figure 1. In brief, first, PMMA foil was treated with oxygen plasma to increase its hydrophilicity for efficient coating of functional materials (Figure 1A, step 1). Then, PEI was inkjet-printed locally on the plasma activated PMMA surface to introduce amine functional groups (Figure 1 A, step 2). The amino-terminated surface is later converted into an aldehyde group by a reaction with glutaraldehyde (Figure 1A, step 3). This step was followed by deposition of bio-ink that contained a capture antibody, precisely onto the aldehyde-functionalized surface, where amino residues of the printed capture antibody form a covalent bond with an aldehyde group (Figure 1A, step 4). Finally, the antibody-patterned PMMA substrates were integrated with R2R hot embossed microfluidic channels by solvent bonding lamination (Figure 1 B). C-reactive protein, a general inflammation and cardiac marker, was used as a model biomarker to evaluate the performance of the antibody immobilization method. According to our results, patterning of the capture antibody in microfluidic platform via covalent linkage significantly improved the antibody binding efficiency and assay signal compared to immobilization by passive binding. With this printed immunoassay biochip, dose-dependent and highly repeatable CRP detection was presented.

## Experimental

### Material and Reagents

Poly (methyl methacrylate) foils (PMMA Plexiglas 99524, thickness 125 µm and 375 µm) were obtained from Evonik Rohm, Germany. Polyethyleneimine (PEI; MW 75 000) was purchased from Sigma Chemicals (St. Louis, MO). CRP antigen (analyte) was purchased from Scripps Laboratories (San Diego, CA), and anti-CRP Mab 6404 (capture antibody) and anti-CRP Mab 6405 (secondary antibody) were obtained from Medix Biochemical (Kauniainen, Finland) and reconstituted in phosphate buffer (pH 7.4) and stored at 4°C until use. Secondary antibody was labeled with Alexa Fluor 546 using Alexa Fluor 546 protein labeling kits (Invitrogen Corp. San Diego, CA), and stored at 4°C until use in aluminum foil-wrapped plastic vials to protect from light. Glutaraldehyde (50% in water), Tween-20, cold water fish gelatin and bovine serum albumin (BSA) were purchased from Sigma-Aldrich (St. Louis, MO).

### Microfluidic Chip Fabrication by Roll-to-Roll Hot Embossing

Microfluidic chips containing 8 parallel straight channels (400 µm wide, 50 µm deep, and 60 mm long) with 12 mm spacing between the individual channels were fabricated by R2R hot embossing on a PMMA polymer sheet with a web width of 200 mm and a thickness of 375 µm. R2R hot embossing was performed by a pilot printing machine fitted with an embossing module. The embossing shim was fabricated on large area, flexible steel (200 mm×400 mm) with a thickness of 110 µm by a wet-etching technique and closed into a cylindrical sleeve by laser welding to form a microstructured embossing cylinder. Hot embossing of the PMMA web was made by continuous feeding of the polymer web between the oil-heated, microstructured embossing cylinder and a counter pressure cylinder. The embossing cylinder temperature was maintained at 105°C and the counter cylinder was pressed with the embossing cylinder to generate a pressure of 20 bars to create the microchannel on the heated PMMA polymer. The resulting channel profiles were characterized by stylus profilometry (Dektak 150, Veeco, USA). The detailed procedure for R2R hot embossing of PMMA foil by an in-house printing machine was reported previously [31].

### Preparation and Characterization of PEI Ink

Polyethyleneimine (PEI) inks were prepared by diluting PEI of molecular weight of 750-kDa **(**50% w/w aqueous solution) with PBS (phosphate buffer) to a final concentration of 0 to 6% (w/w) followed by adjusting the pH to 11.5 by addition of 1 M NaOH to increase the efficiency of PEI attachment to the PMMA surface [29]. The dynamic viscosity of the inks was determined by a rotational viscometer (Brookfield Rheometer LV, Germany) at 23°C by varying the shear rate between 0 to 5000 s^−1^. The viscosity value for each measured solution was reported at a shear rate of 2500 s^−1^. The surface tension of the inks was measured by a CAM 2000 optical contact angle meter (KSV Instruments Ltd., Helsinki, Finland). To examine the effect of PEI concentration on the binding capacity of the capture antibody, a fluorescence sandwich immunoassay was performed on the PEI-coated PMMA films in a FAST Frame multi slide plate. PMMA slides were inserted into a Whatman FAST Frame multi slide plate containing 16 wells. One hundred microliters of PEI inks (0% to 6%) were added to each well and incubated for 1 hour. Next, 100 µl of 2% glutaraldehyde was added and incubated for 20 minutes. Next, 100 µl of the capture antibody (5 µg/ml in PBS at pH 7.4) was added and incubated for 1 hour. To block nonspecific binding, 150 µl of PBS containing 2% BSA and 0.2% fish gelatin was added to each well and incubated for 1 hour. Finally, 50 µl of CRP antigen (1 µg/ml in 1% BSA) and 50 µl of the secondary antibody (0.0132 mg/ml in 1% BSA) were added together to each well and incubated for 1 hour. Between additions of the different solutions, the wells were washed three times with 150 µl of PBST (pH 7.4). The PMMA slides were removed from the FAST Frame multi slide plate and dried under a stream of nitrogen prior to fluorescence measurement.

### Inkjet Printing of PEI

PMMA slides (8 cm×10 cm) were cut from a 125-µm-thick web for inkjet printing. Prior to inkjet printing, the slides were cleaned with 2- propanol and treated with oxygen plasma with a Tep1a 440-G plasma etcher (300 W and 30 s). To avoid clogging of the printhead nozzles, the ink was filtered through a nylon membrane filter with a 0.2-µm pore size. A 800 µm×1 mm pattern of the PEI ink was deposited in a horizontal orientation at discrete positions with 12 mm spacing on PMMA foil by piezoelectric inkjet printing technology (Dimatix DMP-2800 from Fujifilm Dimatix Inc., USA). Immediately after printing, the positions of the patterned areas were marked for subsequent alignment with the capture antibody printing. The PEI patterned PMMA substrates were washed with PBST, rinsed with deionized water and soaked in 2% GA for 20 minutes to generate a reactive aldehyde group for covalent binding of the printed capture antibody. Finally, the surface was washed with deionized water and dried under a stream of nitrogen.

### Inkjet Printing of Antibody

Bio-ink containing 0.5 mg/ml of anti-CRP antibody was prepared in PBS deposition buffer (pH7.4). Tween 20 (0.1% w/w) was added to the deposition buffer as a surface tension modifier and sucrose (10% w/w) was included to protect the antibodies from the denaturing effect of dehydration during the printing process [20]. The bio-ink was filtered using a 0.2-µm membrane filter and deposited using the same printing system that was used for the patterning of PEI ink. The bio-ink was overprinted on the PEI-GA patterned PMMA substrate with a size of 1.5 mm×1.5 mm to cover the entire 800 µm×1 mm underlying PEI-GA patterned areas. The bio-functionalized slides were then left for one hour at room temperature and bonded with a PMMA chip containing microchannels by solvent bonding lamination with the aid of an electrically driven metal rod on a control coater machine (K Control Coater 202, R K Print-Coat Instruments Ltd., UK). Briefly, the bio-functionalized PMMA slide was put on top of the microstructured PMMA substrate and fixed by a piece of scotch tape after insuring that each patterned area covered the corresponding microchannel. The upper PMMA slide was bent upwards, and 200 µl of a solvent mixture composed of ethyl acetate and isopropanol (35∶65%v/v) was pipetted between the two substrates. Immediately, the metal rod was driven at a speed of 16 cm/s. Immediately after solvent bonding laminations, the excess solvent was removed from the channels by sucking slowly with a micropipette tip connected to a water aspirator tube.

### Surface Characterization

AFM observation was made in tapping mode using a Veeco Dimension 3100 AFM system (Veeco Metrology Group, Santa Barbara, CA) to investigate the morphology of inkjet-printed PEI and antibody films on PMMA. A 2% (w/w) PPMA/toluene solution was prepared by dissolving PMMA foil in toluene and filtering through a 0.2-µm membrane filter. The filtered solution was spin-coated on a silicon wafer at speed of 1500 rpm for 30 seconds, and spin- coated films were subsequently cured for 48 hours at 100 ^o^C under vacuum. The cured PMMA films were treated with oxygen plasma, and patterned with PEI polymer and activated with GA followed by antibody deposition. AFM imaging was performed under ambient conditions using a commercial silicon microcantilever probe (NSC14; µ-Masch, Estonia) with a resonance frequency of 160 kHz. The manufacturer’s values for the probe tip radius and probe spring constant were 10 nm and 5.7 N/m, respectively. The reported root-mean-square (RMS) roughness value for each surface was an average of three images scanned at different locations on the same surface to insure a large-scale spatial averaging of the surface roughness.

To investigate the surface elemental compositions of PMMA foils before and after inkjet printing of PEI, X-ray photoelectron spectrophotometer (XPS) measurements were performed with a high resolution electron spectrometer (AXIS 165 by Kratos Analytical) using monochromatic Al Ka irradiation at 100 W and soft neutralization with slow thermal electrons. Survey scans were recorded with 1 eV step and 80 eV pass energy, and the high resolution C 1 s, O 1 s and N 1 s regions were analyzed using 0.1 eV step and 20 eV pass energy. Each sample was measured at three locations with an analysis spot of 1 mm^2^. The data were collected at 90° from the sample surface.

### Sandwich Immunoassay on a Microfluidic Chip

Fluorescence immunoassay was used for the detection of CRP antigen in the microfluidic chip. Prior to the assay, PBS-buffer was prepared by dissolving 1.44 g Na_2_HPO_4_, 0.211 g NaH_2_PO_4_, and 8 g NaCl in 1000 ml of deionized water and the pH was adjusted to 7.4 by adding 1 M HCl. The washing buffer (PBST) contained PBS and 0.5% Tween 20. The blocking reagent contained 2% BSA, 0.2% fish gelatin and 0.1% Tween 20 in PBS buffer (pH 7.4). Standard solutions containing 0 to 1500 ng/ml CRP antigen and 0.0132 mg/ml detecting antibody at final concentrations were prepared in PBS buffer containing 1% BSA (pH 7.4). Before the assay the laminated microchips were washed with 10 µl of PBST. Next, 10 µl of the blocking reagent was introduced and incubated for 20 min. Subsequently, 5µl of each standard was loaded into the microchannels and incubated for 15 min. Between additions of the above reagents in the microchannels, washing was performed with 10µl of PBST. Samples and reagents were pipetted to the sample port of the microchips, and the microchannels were filled by capillary-driven flow. Between immunoassay steps, the microchannels were emptied with a syringe.

### Fluorescence Imaging and Data Analysis

Fluorescent PMMA plates and microfluidic channels were scanned using a Typhoon 9410 fluorescent imager (GE Healthcare) in fluorescence acquisition mode with 100-µm pixel size and using the same photomultiplier (PMT) voltage of 450 V. A Neodymium: Yttrium Aluminum Garnet (Nd:YAG) 532-nm solid-state laser was used as the excitation source and fluorescence emission signal was collected through a 580/30 nm band pass filter. The digitalized fluorescence image of each analyzed sample was displayed and quantified using the Image Quant TL analysis system (Amersham Biosciences). Microscopic fluorescence images were captured using an Axion software package (Carl Zeiss Microimaging GmbH, Jena, Germany) under a fluorescent microscope (Carl Zeiss Microimaging GmbH). Statistical analysis was performed, and the significant difference between two groups was evaluated by the student’s t-test; the level of significance was set as P<0.05.

## Results and Discussion

### PEI Polymer Ink Formulation

**Figure 2:**
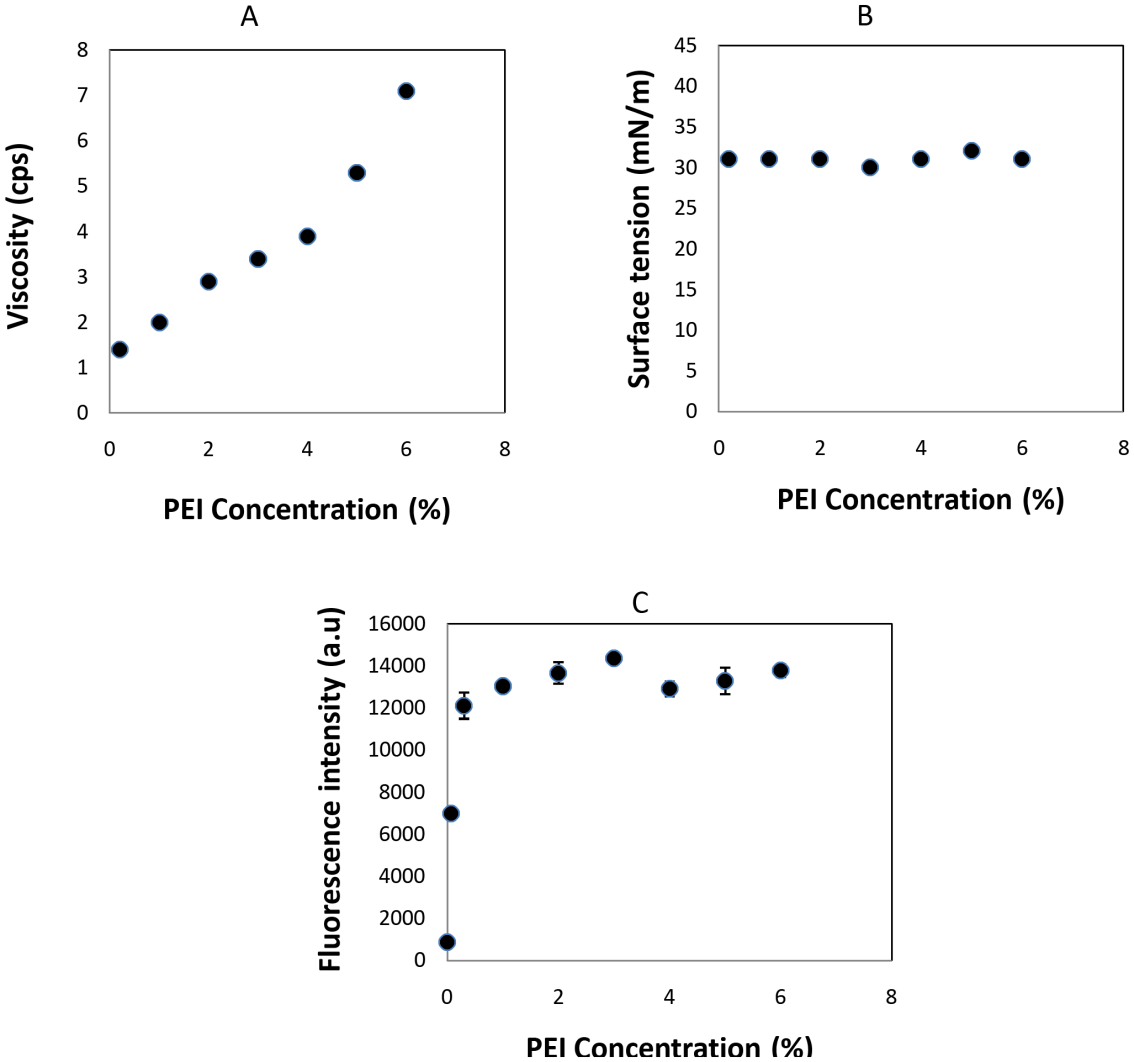
Basic properties of PEI inks at different polymer concentrations. (A) Viscosity, (B) surface tension, (C) effect of PEI concentration in the ink on anti-CRP antibody binding capacity of PMMA substrate (5µg/ml anti-CRP, 500 ng/ml CRP, 0.0132 mg/ml secondary antibody; n = 2).

The physicochemical properties of a polymer ink such as viscosity and surface tension have significant effects on its inkjet printability, pattern accuracy and pattern resolution [32]. Therefore, the PEI ink was optimized by monitoring its viscosity and surface tension at various PEI concentrations to insure stable drop formation. The ink viscosity should be below 20 cps at least and ideally below 10 cps, in piezoelectric inkjet printing systems [32], [33]. At higher viscosities, ink drop formation can be hindered because the force generated by a piezoelectric inkjet printer is limited [34]. The presence of PEI in the ink significantly increased its viscosity from 1.2 cps at 0.1% to 7 cps at 6% (Fig. 2A). The surface tension of the ink for piezoelectric inkjet printing should be in the range of 30–70 mN/m, which is high enough to prevent dripping of the ink on the nozzle and low enough to spread over the substrate [32]. The surface tension of the initial aqueous solution of PEI was lowered from 65 mN/m to 31 mN/m by addition of 0.1% (w/w) Tween 20 to increase the wetting and spreading of the ink droplets on the surface of the PMMA substrate. As seen in Fig. 2B, the presence of the PEI polymer at different concentrations did not affect the value of the surface tension of the ink containing 0.1% Tween 20. The surface tension values of the PEI inks were about 31 mN/m regardless of the PEI concentration. The effect of the PEI concentration in the ink on the capture antibody binding capacity was studied systematically by fluorescence sandwich immunoassay made on PMMA film coated with inks containing 0% to 6% (w/w) PEI polymer and 0.1% (w/w) Tween20. Increments of the PEI concentrations from 0 to 0.1% resulted in a corresponding increase in fluorescence intensity (Fig. 2C). However, at PEI concentrations above 0.1% (w/w), the fluorescence intensity began to plateau. This finding could be because of the removal of the excess amount of adsorbed PEI during the washing step.

All of the studied inks containing 0.2% to 3% of PEI could be inkjet-printed after adjusting the printer parameters. However, printing of a polymer solution containing higher than 0.2% of PEI resulted in the removal of excess deposited PEI and a noticeable attachment of it to the surrounding predesigned pattern area during the first rinse. This removal was evidenced by high nonspecific adsorption of biomolecules outside the desired pattern area during the assay. Therefore, ink containing 0.2% of PEI and 0.1% Tween 20 (pH 11.5) was used as suitable polymer ink for patterning of PMMA polymer foil.

### PEI Patterning on PMMA Foil and Surface Characterizations

Surface properties of PMMA such as low surface energy and hydrophobicity make it difficult to be wetted and coated with other functional materials [26]. Hence, oxygen plasma activation was first performed to improve its wettability characteristics for efficient attachment of the printed PEI. After plasma activation of PMMA foil, PEI ink was inkjet-printed locally for subsequent patterning of antibody. The printed PEI film showed good adherence to the PMMA surface when exposed to aqueous media as observed from protein binding analysis after surface activation with glutaraldehyde. Despite the fact that the drying time for the printed polymer solution is only a few seconds, the deposited PEI film was not observed to be delaminated from the surface of PMMA during washing with water even immediately after printing. This property resulted in a fast and straightforward method to generate a local aldehyde surface on the PMMA substrate for covalent patterning of biomolecules.

**Figure 3:**
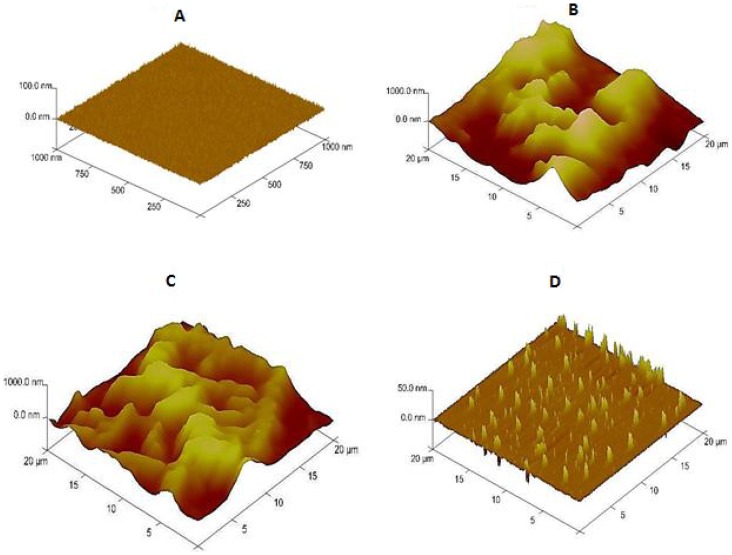
AFM images of (A) native PMMA (1 µm×1 µm) (B) PEI patterned native PMMA (20 µm×20 µm) (C) PEI patterned plasma treated PMMA (20 µm×20 µm) (D) antibody patterned PEI-GA film on plasma treated PMMA (20 µm×20 µm).

AFM microscopy was used to investigate the effect of oxygen plasma activation of the PMMA foil on morphology of the printed PEI film. Figure 3 compares AFM images of the different PMMA films in the tapping mode. The spin coated PMMA film on a clean silicon wafer (Fig. 3A) exhibited flat morphology with a root-mean-square (RMS) roughness value of 2.1±0.7 nm. For PEI film printed on native (Fig. 3B) and oxygen plasma-activated (Fig. 3C) PMMA surfaces, the RMS values were 1.6±0.4 µm and 0.9±0.1 µm, respectively. This indicates that the film formed on plasma activated PMMA surface was smoother because PEI polymer droplets spread to all surface holes providing a continuous and homogenous film surface for effective immobilization of the printed biomolecules. The surface roughness of the PEI film was further decreased to a value of 35.2±3.1 nm after activation with GA and deposition of bio-ink containing capture antibody (Fig. 3D).

**Figure 4:**
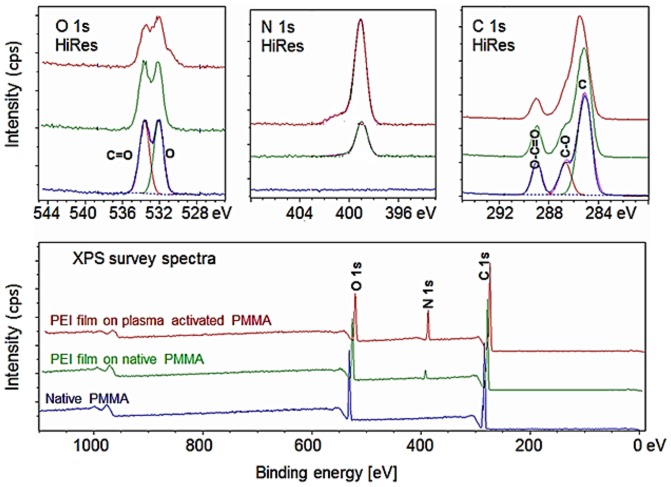
XPS spectra of native PMMA, PEI patterned native PMMA and PEI patterned plasma activated PMMA surface.

Quantitative surface elemental composition of native PMMA foil (control), PEI film on native PMMA, and PEI film on an oxygen plasma-activated PMMA surface were analyzed by X-ray photoelectron spectroscopy (XPS) to investigate the effect of plasma activation on the printed PEI attachment efficiency. The survey scan of the PEI patterned PMMA films showed three peaks at 285 eV, 399 eV and 532 eV corresponding to C1s, N1s and O1s, respectively (Fig. 4) and were used to estimate the elemental composition of the different PMMA surfaces (Table 1). As expected, no nitrogen entity was detected on the surface of the native PMMA foil (control sample) with a method having a detection limit of 0.2% mass fraction. As seen from Fig. 4, plasma activation of PMMA foil prior to inkjet printing of PEI effectively increased the total mass fraction of nitrogen compared to printing on native PMMA foil. The nitrogen mass fraction on the PEI surface on native PMMA was 4.1% while this magnitude increased to 12.5% when plasma activation of PMMA surface was performed prior to inkjet printing of PEI. The nitrogen mass fraction difference on the two PMMA surfaces is statistically significant (P<0.05). This result was consistent with a previously reported result where the amount of PEI adsorption from aqueous solution onto a PMMA surface was indirectly monitored by ζ potential measurement [35]. Furthermore, as shown in Table 1 the nitrogen mass fraction associated with amide bond (O = C-N) is 0.4% for PEI film printed on native PMMA surface while this magnitude is 1.8% when PEI is printed on oxygen plasma activated PMMA surface. Also, the decrease in oxygen mass fractions with increasing PEI attachment is in agreement with molecular structure of PEI as it contains only nitrogen, carbon and hydrogen. Plasma activation incorporates several negatively charged oxygen-based functional groups at the outer most surface of PMMA [26]. Additionally, as PEI is a polycationic polymer [30], the plasma treated PMMA surface supports electrostatic attachment between charged amino groups possibly present on the printed PEI surface and the negatively charged plasma-treated surface. Therefore, the stable printed PEI film on the oxygen plasma treated PMMA surface would be provided by the cooperative attachment based on the covalent binding and the electrostatic interaction.

**Table 1:** Mean (±SD)^a^ values of mass fractions of elements on native, PEI patterned native and PEI patterned oxygen plasma treated PMMA surface.

Elemental species	Binding energy (eV)	Native mass conc. (%)	PEI mass conc. (%)	Plasma–PEI mass conc. (%)
C 1s (C-C)	284.5	45.0±1.0	48.5±0.5	49.3±2.0
C 1s(O-C)	286.0	17.4±0.4	15.2±0.4	13.8±2.8
C 1s(O-C = O)	288.4	15.7±0.9	13.7±0.2	10.1±0.8
C 1s (Total C)		78.1±1.0	77.2±0.8	73.2±0.9
O 1s(C-O-C)	531.5	10.5±0.1	9.6±0.2	7.7±0.4
O 1s(C = O)	533.0	10.8±0.2	9.4±0.1	6.4±0.8
O 1s (Total O)		21.9±0.9	19.1±0.3	15.3±0.8
N 1s (C-N)	399.0	nd^b^	3.6±0.6	10.7±1.3
N 1s(O = C-N)	400.5	nd^b^	0.4±0.1	1.8±0.1
N 1s(Total N)		nd^b^	4.1±0.5	12.5±0.8

a mean and SD were calculated from three measurements on different area of a sample surface, and ^b^not detected with a method with detection limit of 0.2% mass fraction.

### Inkjet Printing of Antibody onto PEI–GA Patterned PMMA Surfaces

The printing down protein immobilization format offers a high-throughput capability, low sample consumption, practicality for manufacturing protein-patterned surfaces and storage [36]. However, maintaining structural conformation and bioactivity of the printed protein is challenging as proteins are susceptible to desiccation-induced damage [20], [36]. Therefore, in this study, 10% sucrose was added to the printing buffer to minimize the denaturing effect of the printing process [20]. The final ink was determined to contain 10% sucrose, 0.5 mg/ml anti-CRP antibody, and 0.1% Tween 20 as a surface tension modifier. The bio-ink exhibited stable and repeatable drop formation during jetting.

Before inkjet printing of the bio-ink, the PEI patterned PMMA surface was incubated in 2% GA solution to generate a reactive aldehyde surface (PEI-GA). These aldehyde groups readily form covalent bonds with the primary amines of the printed capture antibody [23], [37]. However, controlling the antibody orientation is essential for sensitive immunosensor development. Although covalent bonding leads to some loss in antibody activity, it was demonstrated that amine-functionalized surfaces improve the orientation of immobilized IgG molecules [38]. Thus, the positively charged NH_2_ group on the printed PEI molecule that remains unreacted with GA can help to improve the antibody binding efficiency. The activity of the capture antibody also can be further improved through a biotin-avidin affinity immobilization method by printing biotinylated capture antibody on streptavidin coated surfaces [20], [24]. However, extensive incubation of the substrates in streptavidin solution limits the practical applicability of the immobilization method for easy and rapid fabrication of protein-patterned surfaces. Therefore, we used covalent immobilization of antibodies for rapid fabrication of antibody patterned PMMA foil which is to be integrated in microfluidic channel. After printing the bio-ink precisely onto the PEI-GA patterned PMMA surface, the PMMA foil was bonded with R2R hot embossed PMMA microchannel by solvent bonding lamination. A visually uniform and smooth bio-ink film was observed inside the laminated microfluidic platform and the bioactivity of the capture antibody was preserved after solvent bonding lamination.

**Figure 5:**
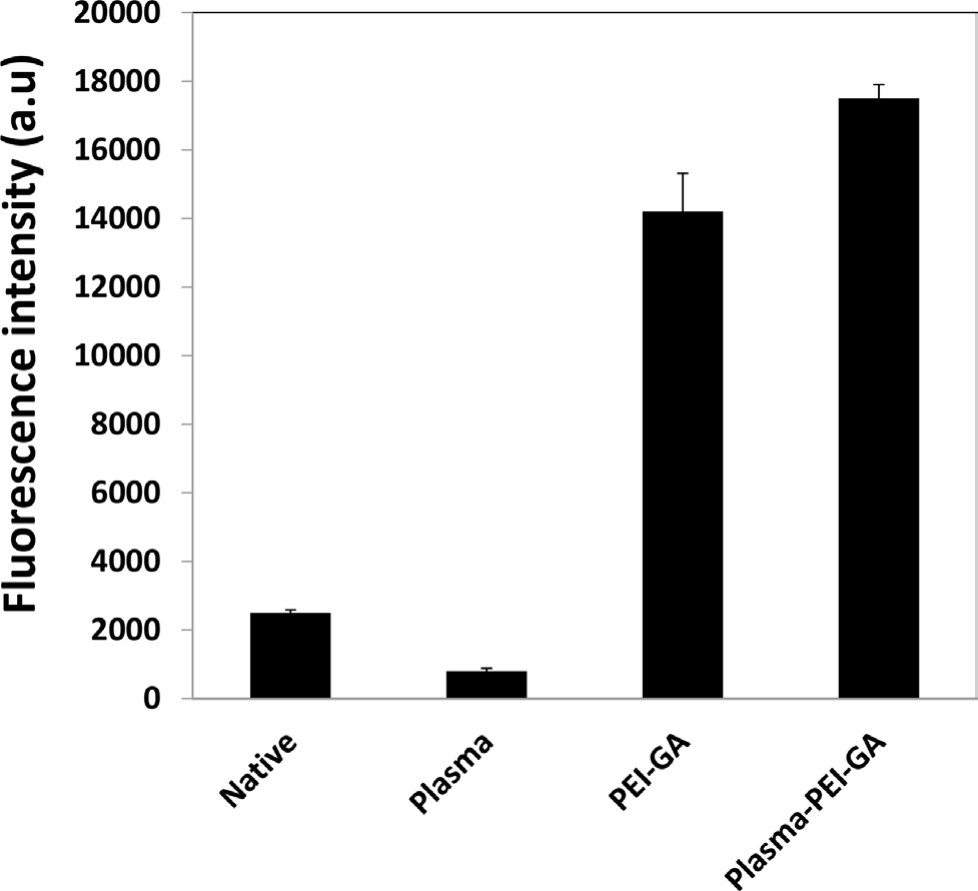
Comparison of performance of biochips for detection of CRP antigen where anti-CRP antibody inkjet printed on PMMA substrate modified under different conditions (500 ng/ml CRP and 0.0132 ng/ml secondary antibody; n = 3).

The binding capacities of the inkjet-printed anti-CRP of the modified and native PMMA substrates were compared. Figure 5 shows the performance of the immunoassay for the detection of 500 ng/ml CRP antigen when anti-CRP was inkjet printed on PMMA substrates functionalized under different conditions. A nine-fold increase was observed in the fluorescence intensity for the CRP recognized by anti-CRP printed on the plasma–PEI-GA patterned PMMA surface compared to the anti-CRP printed on the native PMMA surface. CRP recognition capacity of the printed anti-CRP on the plasma-PEI-GA patterned surface was also higher than the one printed on the PEI-GA patterned surface (P<0.05). The fluorescence intensity of the CRP assay when anti-CRP was printed on plasma treated PMMA was only 5% of the intensity recorded on the plasma-PEI-GA patterned PMMA substrate. These results confirmed that a combination of oxygen plasma treatment of PMMA and inkjet printing of PEI effectively increased anti-CRP binding capacity of PMMA surface, and the subsequent CRP recognition capacity of the biochip.

**Figure 6:**
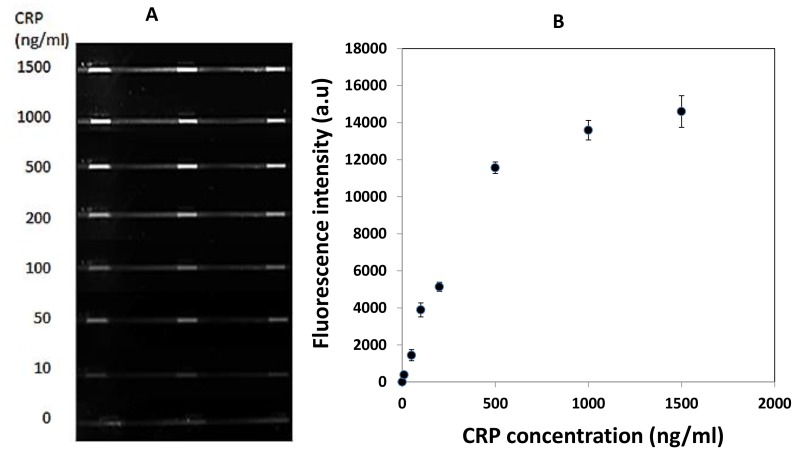
Microfluidic biochip responses for detection of CRP antigen. (A) Fluorescence image of the biochip after immunoassay. Each microfluidic channel was patterned with three replicate CRP capture antibody spots. Samples containing CRP at concentration of 0, 10, 50, 100, 200, 500, 1000, 1500 ng/ml were infused through separate sample channels (rows 1–8, respectively). In each channel the bound antigen was detected with fluorescently labelled secondary antibody (0.0132 mg/ml). (B) Dose response curves for CRP antigen. Fluorescence values were plotted for each CRP concentration. Each data point represents the mean ± SD for the three spots within each microchannel.

Usually the area outside the protein printed regions (background) must be passivated to resist non-specific protein adsorption [39]. However, as shown in Fig. 6A, a highly specific CRP binding to the printed anti-CRP pattern was demonstrated with minimum nonspecific adsorption to the surrounding surface of anti-CRP-patterned regions. The standard deviation in fluorescence intensity of equal small areas measured within each antibody-patterned region was less than 10% of the overall signal (n = 4). This highlights the ability of inkjet printing system to generate a highly homogeneous and reproducible antibody pattern. Therefore, our antibody immobilization approach could be applied for generating high quality, covalently bound and localized antibody pattern on PMMA microfluidic platform for on–chip immunoassays. Furthermore, the antibody patterning approach can be scaled up to incorporate several additional patterned antibodies in a single microchannel for multiplexed assay.

### Detection of CRP on a Microfluidic Chip

The overall performance of the printed antibody in the microfluidic platform was evaluated with fluorescence-based sandwich immunoassay for detection of CRP as a model analyte. Figure 6A shows fluorescence microscope image of the response obtained when a sandwich immunoassay was performed on the biochip for detection of CRP antigen. It was clear that an increase in CRP concentration from 0 ng/ml to 1500 ng/ml resulted in a corresponding increase in the fluorescence intensity. The dose-response curve in Fig. 6B was generated by plotting the mean fluorescence intensity ± standard deviation (SD) for the three anti-CRP spots at each tested CRP concentration. The calculated limit of detection (the value greater than the blank ±3 SD) was determined to be 5.2 ng/ml. The lowest concentration of CRP for which detection was reliably differentiated from background signal was 10 ng/ml. The dynamic range of the biochip assay was from 10 ng/ml to 500 ng/ml. (R^2^ = 0.991). At concentrations above 500 ng/ml, the dose response curve started to plateau. The coefficient of variation in fluorescence among the three detection spot areas within a single microchannel ranged from 4.3% at 10 ng/ml CRP to 9.7% at 1500 ng/ml CRP (Table 2). The variability of fluorescence intensity in three different channels on the same chip ranged from 4.5% at 10 ng/ml CRP to 5.9% at 1500 ng/ml CRP (Table 3). Assays performed on three independent biochips showed a variability of 10% at 500 ng/ml CRP. The results indicated a good level of reproducibility of fluorescence intensity generated by antigen-antibody reaction even in different microchannels.

**Table 2:** Reproducibility of fluorescence intensity of CRP binding to the patterned anti-CRP spots in one channel.

Spot number	CRP concentration (ng/ml)							
	0	10	50	100	200	500	1000	1500
1	2118	2988	3874	7387	9638	15443	17990	17700
2	1949	2777	3593	6504	8863	14020	14774	15937
3	2041	2884	3711	6139	8971	13170	16387	16944
Average	2036	2884	3659	6677	9157	14211	16383	16860
RSD (%)	4.1	3.6	5.2	6.9	4.6	5.2	8.1	9.8

**Table 3:** Reproducibility of fluorescence intensity of patterned anti-CRP spots in three different channels.

Channel number	CRP concentration (ng/ml)							
	0	10	50	100	200	500	1000	1500
1	1951	2638	3413	6178	8419	14319	16815	16512
2	2012	2838	3525	7017	9856	14670	15140	18553
3	1838	2539	3680	5832	8722	12511	17097	17206
Average	1934	2672	3539	6342	8999	13833	16351	17424
RSD(%)	4.5	5.6	3.7	9.6	8.4	6.4	8.3	5.9

According to human-CRP ELISA kit supplier (Invitrogen Corp., CA) product information the inter-assay and intra-assay precisions are between 6.1% at 51 pg/ml CRP to 7.5% at 709 pg/ml CRP and 9.9% at 57 pg/ml CRP to 9.3% at 770 pg/ml CRP, respectively. Thus, the reproducibility of CRP determination on the fabricated biochip is comparable with the ELISA on conventional 96-microtiter plate. The microfluidic CRP assay was not as sensitive as the commercial ELISA methods in conventional 96-well format. However, the sensitivity is comparable with the previously presented microfluidic assay on PDMS substrate (10 ng/ml) [19]. The sensitivity of the CRP determination on the developed microfluidic chip could be further improved with careful optimization of the assay protocol. Generally, the assay on the low cost R2R hot embossed microfluidic channels with inkjet-printed capture antibodies offers some distinct advantages over the conventional immunoassays on microtiter well format. In the present study, the total immunoassay time required for CRP measurement was approximately 35 min while hours is required in ELISA. The total volume of sample required was 5 µl which is much less than the assay in microtiter plates where 100 µl of sample is required. Additionally array-based assay in microchips combined with fluorescence detection is becoming one of the most powerful tools for rapid multiplexed protein analysis [15], [20], [40]. In contrast, conventional ELISA suffers from relatively low throughput because of its lack of multiplexing ability and high reagent and sample consumption [20], [41]. The method outlined in this study demonstrated the fabrication of low cost and disposable polymer based immunoassay chips by fully inkjet printing of all the necessary reagents for covalent patterning of capture antibodies. The technique is time-efficient and cost-effective and can be used for large scale fabrication of biochips using advanced printing systems and automation.

### Conclusions

An efficient and versatile technique for patterned immobilization of functional antibodies within polymer-based microfluidic platform fabricated by R2R hot embossing has been introduced. The antibody patterning approach consisted of inkjet printing PEI on a plasma-activated PMMA foil surface, which was later activated with GA to generate an aldehyde terminated surface for covalent binding of biomolecules, and printing a functional antibody precisely onto the well-defined PEI-GA pattern. Anti-CRP antibody was patterned on PMMA foil as a model capture antibody and the patterned PMMA foil was bonded with R2R hot embossed PMMA microchips by solvent bonding lamination. The patterned anti-CRP antibody in the sealed microfluidic channel remained active and showed dose-dependent bioactivity for CRP antigen with a detection limit of 5.2 ng/ml and a dynamic range from 10 ng/ml to 500 ng/ml (R^2^ = 0.991). As a result, the method can be applied for the fabrication of low cost foil-based microfluidic immunoassay biochips for POCT application using an inkjet printing technique. In comparison with dip-coating antibody immobilization techniques, the method minimizes reagent consumption and drastically reduces repetitive washing and incubation steps. As a result, this method provides an advantageous option for mass fabrication of disposable microfluidic-based immunoassay biochips using advanced printing systems and automation. The developed method is highly versatile and can be scaled up to incorporate several additional patterned capturing proteins in a single microfluidic channel for multiplexed immunoassays.
